# Delivery of health care at the end of life in cancer patients of four swiss cantons: a retrospective database study (SAKK 89/09)

**DOI:** 10.1186/1471-2407-14-306

**Published:** 2014-05-01

**Authors:** Klazien W Matter-Walstra, Rita Achermann, Roland Rapold, Dirk Klingbiel, Andrea Bordoni, Silvia Dehler, Gernot Jundt, Isabelle Konzelmann, Kerri M Clough-Gorr, Thomas D Szucs, Matthias Schwenkglenks, Bernhard C Pestalozzi

**Affiliations:** 1 Institute of Pharmaceutical Medicine (ECPM), University of Basel Basel, Switzerland; 2 Swiss Group for Clinical Cancer Research (SAKK) Bern, Switzerland; 3 Helsana Group, Zürich, Switzerland; 4 Cancer Registry Ticino Locarno, Switzerland; 5 Cancer Registry Zürich and Zug, University Hospital Zürich Zürich, Switzerland; 6 Cancer Registry Basel-Stadt and Basel-Land, University Hospital Basel Basel, Switzerland; 7 Cancer Registry Valais Sion, Switzerland; 8 National Institute for Cancer Epidemiology and Registration (NICER) Zürich, Switzerland; 9 Institute of Social and Preventative Medicine (ISPM), University of Bern Bern, Switzerland; 10 Division of Oncology, University Hospital Zürich Zürich, Switzerland

**Keywords:** Cancer, End-of-life, Radiotherapy, Chemotherapy, Health insurance, Hospitalization

## Abstract

**Background:**

The use of cancer related therapy in cancer patients at the end-of-life has increased over time in many countries. Given a lack of published Swiss data, the objective of this study was to describe delivery of health care during the last month before death of cancer patients.

**Methods:**

Claims data were used to assess health care utilization of cancer patients (identified by cancer registry data of four participating cantons), deceased between 2006-2008. Primary endpoints were hospitalization rate and delivery of cancer related therapies during the last 30 days before death. Multivariate logistic regression assessed the explanatory value of patient and geographic characteristics.

**Results:**

3809 identified cancer patients were included. Hospitalization rate (mean 68.5%, 95% CI 67.0-69.9) and percentage of patients receiving anti-cancer drug therapies (ACDT, mean 14.5%, 95% CI 13.4-15.6) and radiotherapy (mean 7.7%, 95% CI 6.7-8.4) decreased with age. Canton of residence and insurance type status most significantly influenced the odds for hospitalization or receiving ACDT.

**Conclusions:**

The intensity of cancer specific care showed substantial variation by age, cancer type, place of residence and insurance type status. This may be partially driven by cultural differences within Switzerland and the cantonal organization of the Swiss health care system.

## Background

Several studies in the United States and Europe have shown that the use of anticancer treatments at the end-of-life has increased considerably [1-4]. To a substantial extent, treatment patterns seem to depend on medical as well as nonmedical (hospital type, socio-demographic) factors [5-7]. In addition, studies in health services research have shown that the delivery of health care may be quite unequal between patient groups and/or in different geographic areas, despite existing guidelines and standard procedures [8-10]. Non-cancer related studies for Switzerland have revealed large variations in health care utilization among geographic regions [11-14]. However, to the best of our knowledge, no study on the delivery of health care at the end-of-life of cancer patients has been performed in Switzerland.

The implications of time trends and diversity in treatment patterns at the end of life are unknown. Irrespective of this, the use of anticancer treatments is regarded as an important descriptor of end-of-life care for cancer patients [15-17]. With rising health care costs, ever new expensive anticancer drugs being released and a persistent focus of political attention the necessity to provide independent data on the use of resources at the end of life is self-evident.

The development of large electronic databases by health insurance companies, cancer registries and hospitals during the last decades has facilitated research in this direction considerably [3,18-20]. The combination of claims databases, cancer registries and patient records has previously been used to study time trends in chemotherapy use at the end-of-life [2,19,21]. Swiss cancer registries are organized on a cantonal basis and are lacking in several cantons (2010 data coverage approximately 70% of the national population). Large (national) or smaller regional health insurance companies provide compulsory health insurance in Switzerland.

We have studied patterns of care in recently deceased patients to gain initial insight and provide urgently needed information on current end-of-life care for cancer patients in Switzerland. Data from one large health insurance company were combined with data from four cantonal cancer registries.

Given a lack of published Swiss data, the first main objective of the study was to describe delivery of health care during the last 30 days before death in terms of therapies used and hospitalization frequencies, for all cancers combined and for major cancer types (lung, breast, prostate, colon or hematological cancers). The second main objective was to assess the magnitude and significance of effects of demographic, geographic and insurance coverage-related factors on the above named indicators. The study was not designed for and does not intend to make any value judgments on the appropriateness of the health care provided.

## Methods

### Study population

This retrospective study included patients 20 years or older at time of cancer diagnosis who died between 2006 and 2008, lived in one of the participating Swiss cantons, and were customers of Helsana Group insurance company for at least one year before death. Eligible patients were identified by deterministic linkage of the Helsana health insurance claims data with cancer incidence data from four cantonal cancer registries, Basel (BL/BS), Ticino (TI), Valais (VS) and Zürich (ZH). All data were linked using the SAS® based “The Link King”© software [22]. It was not possible to obtain informed consent from relatives of the deceased patients. Therefore, privacy protecting linkage procedures were utilized and all patient data was anonymized. The study was approved by the ethics committees of the BL/BS (Ethikkommission beider Basel), TI (Comitato etico cantonale), VS (Commission Cantonale Valaisanne d’Ethique Médicale) and ZH (Kantonale Ethikkommission Zürich) and by an expert committee responsible for data protection issues at the Swiss Federal Office of Public Health.

### Data sources

#### *Helsana insurance claims*

The Helsana Group (http://www.Helsana.ch) is one of the largest Swiss health insurance companies and provided health insurance to 1,28 million customers (about 20% of the Swiss population) in 2006. Health insurance is compulsory in Switzerland for every resident and is provided by up to 90 different insurance companies on a non-profit basis. It covers events of general medical illness and pregnancy. Federal law uniformly defines the reimbursement package. Through voluntary supplementary health insurance, coverage for additional health care services can be obtained. Unlike benefits from compulsory insurance, benefits from supplementary health insurance differ depending on the product chosen. Supplementary insurance can be purchased at the same or another health insurance company. In our study we only took into account the services covered by the compulsory health insurance. The Helsana data provided detailed information on all outpatient medical services provided. For inpatient services no such details were available. The Helsana database is not publicly available and permission to use the data was given by the Helsana directorate and approved by the above-listed ethics committees and expert committee.

#### *Cancer registry incidence data*

In Switzerland there is no national cancer registry and only 14 out of 26 cantons had a cantonal cancer registry in 2010. All cantons with a cancer registry were contacted by the National Institute for Cancer Epidemiology and Registration (NICER) and asked to take part in the study. The registries of four cantons agreed to participate: BS/BL (urbanization rate 90%, language German, one university hospital), TI (urbanization rate 82%, language Italian, no university hospital), VS (urbanization rate 53%, language German and French, no university hospital) and ZH (urbanization rate 90%, language German, one university hospital) [23]. The cancer registries provided information on cancer diagnosis (ICD-10) and the exact date of death.

#### *Hospital data*

Swiss claims data relating to inpatient episodes in acute care hospitals do not contain sufficient detail on the treatments or diagnostic procedures performed. Therefore, this information was collected from patient records in the treating hospitals for all patients with a hospital stay during the last 30 days before death (including those who were admitted before day 30 before death but discharged within 30 days before death).

### Outcomes and covariates

Primary endpoints of this study were indicators of the intensity of care delivered to cancer patients in the last 30 days before death, defined as hospitalization rate, administration of any in- or out-patient ACDT (for definition see Additional file 1: Table S1. Anti-cancer drug therapy medication), administration of any in- or out-patient RT and any cancer related therapy (ACDT and/or RT). These endpoints were set in relation to several potential explanatory variables. These included patient characteristics, such as birthdate (source Helsana), death date (source cancer registries), gender (source Helsana), cancer diagnosis (source cancer registries) and type of health insurance (source Helsana), as well as geographic characteristics, such as canton of residence (source Helsana) and borough type (source Federal Office of Statistics, Helsana).

For prescription drugs, anatomical therapeutic chemical (ATC) codes were available [24]. Outpatient diagnostic tests and therapies were coded according to TARMED, the Swiss tariff system for medical services provided to outpatients [25]. A separate coding system existed for laboratory tests (http://www.bag.admin.ch/al). The date of each outpatient test and treatment were known. For some claims, not all details were electronically accessible but scanned copies of these invoices were available and were reviewed from an electronic archive. All information on ACDT, RT or diagnostic tests performed during the last 30 days before death was recorded. The same information was retrieved from patient records for those patients with a hospitalization within the last 30 days.

Cancer diagnoses were grouped into six groups according to the International Statistical Classification of Diseases and Related Health Problems 10th Revision (ICD-10) codes: colon (ICD-10 = C18.x), hematologic (ICD-10 = C81.x – C96.x), lung (ICD10 = C34.x), breast (ICD-10 = C50.x), prostate (ICD-10 = 61.x) and all others combined (to obtain a meaningful group size). Based on Helsana data, information on supplementary insurance for hospitalization (hospital supplementary insurance HSI) was categorized into three categories. These were obligatory health insurance only (i.e. no HSI patients can only be hospitalized on a general ward in predefined hospitals in their canton of residence, exception only when a certain service is not available in the canton of residence); basic supplementary hospital insurance (ECO) with free choice of hospital all over Switzerland (general ward only); and semi-private or private supplementary hospital insurance (SP + P) with free choice of hospital all over Switzerland and coverage of the additional cost for a double or single room. Two urbanization types for boroughs were used: city (including agglomeration) and rural, as defined by the Swiss Federal Office of Statistics.

### Reason for hospitalization

For all included patients hospitalized during the last 30 days before death, the reason for hospitalization was defined as cancer related (CRH) or non-cancer related (NCRH). CRH included patients who had a primary admission diagnosis indicative of cancer, and/or had cancer related symptom(s) or diseases, or had a non-cancer related reason of admission but had an ongoing active cancer as described in the patient history. NCRH included patients where the diagnosis of cancer was mentioned in the patients’ medical history but without an indication of currently active disease. Cause of death information was not systematically available for all included patients.

### Power

Power calculation for the primary endpoints have considered a range of scenarios for the design parameters of a logistic regression: the expected odds ratio (OR), the percentage of patients reaching the endpoint of interest (E) in the reference class (for example canton = ZH), and the squared multiple correlation coefficient ρ^2^_1.23…*p*_ also known as R^2^[26] when the main variable (canton) is regressed on the other independent variables in the regression model. For most scenarios (e.g. for OR ≥ 1.5, E ≥ 20%, and R^2^ ≤ = 0.2) the expected power was ≥ 80%.

### Statistical analysis

Endpoints, patient characteristics and potential covariates were described using frequencies and percentages in the case of categorical variables. For continuous variables, mean, standard deviation and range were used. The impact of age on the endpoint variables was primarily assessed using age groups (<45, 45-49, 50-54 etc. up to >94 years [27]), in order to detect non-linear associations. Where such non-linear associations were detected, age was divided into splines based on the segmented polynomials approach [28,29].

Multivariate logistic regressions were performed using a stepwise method to select statistically significant explanatory variables. For consideration to enter the model as a co-variant univariate a *P*-value threshold of <0.1 had to be reached. To stay in the multivariate model a threshold of *P* <0.05 was set. The following variables were tested in the model: age at death (using age splines where required), gender, cancer type (colon, hematologic, lung, breast, prostate and other), insurance status (no HSI, ECO, SP + P), canton of residence (BS/BL, VS, TI, ZH), and borough type (urban, rural). For the outcomes of ACDT, RT and ACDT and/or RT, a covariate representing reason for hospitalization (comprising the categories no information available (NoInf)), CRH, NCRH) was added to the model as a technical control variable. In addition, all possible interactions between these variables were tested. Goodness of fit of logistic regression models was tested with the Hosmer and Lemeshow Goodness-of-Fit Test [30]. Parameter estimates and OR were calculated including Walds 95% confidence intervals (95% CI). *P*-values were considered significant if < 0.05, two-sided. Given the explorative nature of this study, there was no adjustment for multiple testing. All statistics were performed with SAS®, version 9.2.

## Results

### Cancer patient identification and inclusion

**Figure 1:**
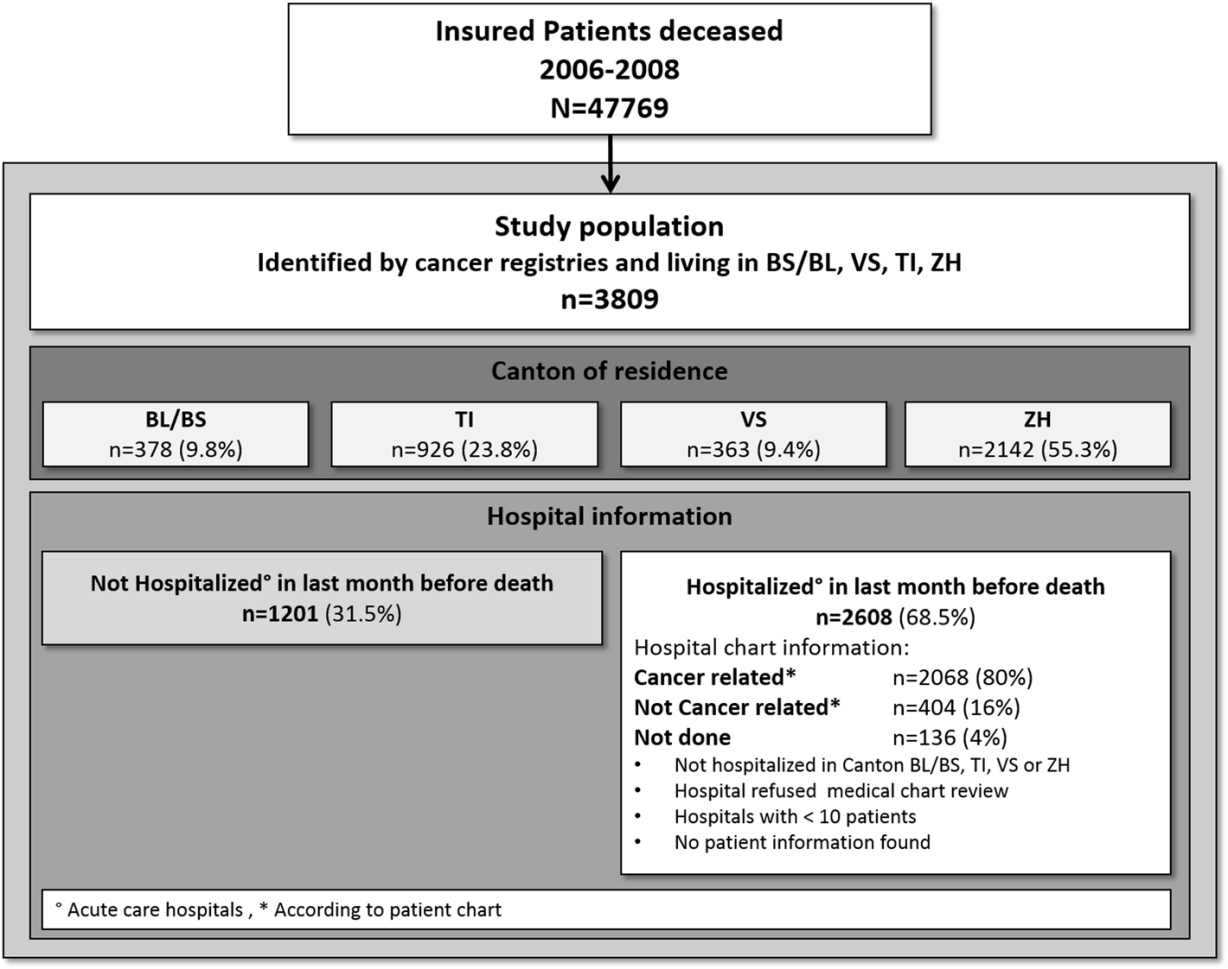
Study patients.

Between 2006 and 2008 the Helsana database contained 47,769 deceased customers who had been insured for at least 1 year before death. After linking these persons to the four cancer registries 3,809 patients were identified as being eligible and were included in the study (see Figure 1). The distribution of patients over the four cantons differed slightly from the expected distribution calculated according to the cause of death statistics (http://www.bfs.admin.ch) and the percentage Helsana insured population (BL/BS = 9.8% expected 12.2%, TI = 23.8% expected 17.2%, VS = 9.4% expected 8.8%, ZH = 55.3% expected 61.8%, see Table 1).

**Table 1:** Descriptive statistics of demographic and geographic information

		**All**	**BS**/**BL**	**TI**	**VS**	**ZH**
**Overall** (expected)		*N* = 3809 (2869)	*N* =378 (350) 9.8% (12.2%)	*N* =926 (493) 23.8% (17.2%)	*N* =363 (252) 9.4% (8.8%)	*N* =2142 (1773) 55.3% (61.8%)
Gender	Male	52.7%	57.1%	55.0%	55.4%	50.4%
	Female	47.3%	42.9%	45.0%	44.6%	49.6%
Cancer diagnosis	Colon	7.9%	8.5%	7.8%	5.5%	8.3%
	Hematologic	6.7%	4.8%	9.1%	1.4%	6.9%
	Lung	14.6%	14.6%	13.3%	17.1%	14.8%
	Breast	9.9%	9.3%	8.3%	7.2%	11.2%
	Prostate	10.4%	7.9%	9.1%	8.3%	11.7%
	Other	50.5%	55.0%	52.5%	60.6%	47.1%
Hospital supplementary insurance status	No HSI	32.1%	35.7%	26.5%	38.8%	32.8%
	ECO	39.9%	34.9%	45.0%	50.2%	36.8%
	SP + P	28.0%	29.4%	28.5%	11.0%	30.4%
Borough type	City + Agglomeration	91.9%	95.8%	89.1%	65.0%	97.0%
	Rural	8.1%	4.2%	10.9%	35.0%	3.0%
Age	Mean	75.5	75.3	76.8	76.2	74.8
	Median	77.3	77.0	78.6	78.0	76.8
	SDEV	12.2	11.6	11.8	12.5	12.3
	Range	23-102	40-102	25-101	29-102	23-102
Time from first diagnosis until death (years)	Mean	4.15	4.60	3.02	4.64	4.49
	Median	2.0	2.0	2.0	2.0	2.0
	SDEV	5.28	6.17	5.08	5.98	5.76
	Range	<1 year-27	<1 year-27	<1 year-12	<1 year-19	<1 year-27

Legend: Hospital supplementary insurance status: no *HSI* = no hospital supplementary insurance, ECO = basic hospital supplementary insurance, SP + P = semi private and private hospital supplementary insurance (2 or single bed room), SDEV = standard deviation.

### Hospital in-patient data collection

During the last 30 days before death 2,608 (68.5%, see Table 2) of the patients were hospitalized in 49 different acute care hospitals. Data collection for in-patient resource use was done in 37 hospitals, 3 hospitals refused chart review and 9 hospitals were not contacted because they would only have provided information on less than 10 patients. Overall, in-patient information was available for 2,494 (96%) of the hospitalized patients. Of these patients, 2,086 (83.6%) had a cancer related hospitalization.

**Table 2:** Descriptive statistics of clinical information

**During last month before death**		**Hospitalized n/% (95% CI**)	**Cancer drug therapy n/% (95% CI**)	**Radiotherapy n/% (95% CI**)	**Cancer drug and**/**or radiotherapy n/% (95% CI**)
	ALL (n = 3809) Died in Hospital (n = 2327, 61.1%)	2608/68.5 (67.0 – 69.9)	552/14.5 (13.4 – 15.6)	293/7.7 (6.7 – 8.4)	773/20.3 (19.0 – 21.6)
Gender	Male (n = 2006)	1463/72.9 (71.0 – 74.9)	310/15.4 (13.8 – 17.0)	175/8.7 (7.5 – 10.0)	440/21.9 (20.1 – 23.7)
	Female (n = 1803)	1145/63.5 (61.3 – 65.7)	244/13.5 (12.0 – 15.1)	114/6.3 (5.1 – 7.4)	334/18.5 (16.7 – 20.3)
Cancer diagnosis	Colon (n = 301)	194/64.5 (59.0 – 69.9)	43/14.3 (10.3 – 18.2)	19/6.3 (3.6 – 9.1)	56/18.6 (14.2 – 23.0)
	Hematologic (n = 255)	187/73.3 (67.9 – 78.8)	48/18.8 (14.0 – 23.6)	16/6.3 (3.3 – 9.3)	56/22.0 (16.9 – 27.0)
	Lung (n = 557)	424/76.1 (72.6 – 79.7)	113/20.3 (16.9 – 23.6)	75/13.5 (10.6 – 16.3)	173/31.1 (27.2 – 34.9)
	Breast (n = 378)	220/58.2 (53.2 – 63.2)	68/18.0 (14.1 – 21.9)	24/6.3 (3.9 – 8.8)	86/22.8 (18.5 – 27.0)
	Prostate (n = 395)	225/64.6 (59.8 – 69.3)	33/8.4 (5.6 – 11.1)	32/8.1 (5.4 – 10.8)	61/15.4 (11.9 – 19.0)
	Other (n = 1923)	1328/69.1 (67.0 – 71.1)	249/12.9 (11.4 – 14.4)	123/6.3 (5.3 – 7.4)	340/17.7 (16.0 – 19.4)
Hospital supplementary insurance status	No HSI (n = 1224)	771/63.0 (60.3 – 65.7)	135/11.0 (9.3 – 12.8)	147/6.5 (5.1 – 7.8)	200/16.3 (14.2 – 18.3)
	ECO (n = 1520)	1053/69.3 (67.0 – 71.6)	201/13.2 (11.5 – 14.9)	116/7.6 (6.3 – 9.0)	293/19.3 (17.4 – 21.3)
	SP + P (n = 1065)	782/73.6 (71.0 – 76.3)	218/20.5 (18.0 – 22.9)	93/8.7 (7.0 – 10.4)	280/26.3 (23.6 – 28.9)
Canton of residence	BS/BL (n = 378)	280/74.1 (69.7 – 78.5)	50/13.2 (9.8 – 16.6)	32/8.5 (5.7 – 11.3)	76/20.1 (16.1 – 24.1)
	TI (n = 926)	654/70.6 (67.7 – 73.6)	169/18.3 (15.8 – 20.7)	53/5.7 (4.2 – 7.2)	208/22.5 (19.8 – 25.2)
	VS (n = 363)	212/58.4 (53.3 – 63.5)	31/8.5 (5.7 – 11.4)	21/5.8 (3.4 – 8.2)	48/13.2 (9.7 – 16.7)
	ZH (n = 2142)	1462/68.3 (66.3 – 70.2)	304/14.1 (12.7 – 15.6)	183/8.5 (7.3 – 9.7)	441/20.6 (18.9 – 22.3)
Borough type	City + Agglomeration (n = 3501)	2419/69.1 (67.6 – 70.6)	515/14.7 (13.4 – 16.0)	226/7.6 (6.6 – 8.4)	718/20.5 (19.1 – 21.8)
	Ruraly (n = 308)	189/61.4 (55.9 – 66.8)	37/12.0 (8.4 – 15.6)	23/7.5 (4.5 – 10.4)	57/18.5 (14.2 – 22.8)
Patient hospitalization/chart information	No information* (n = 1337)		79/5.9 (4.6 – 7.2)	45/3.4 (2.4 – 4.3)	116/8.7 (7.2 – 10.2)
	Cancer related hosp. (n = 2068)		467/22.6 (20.8 – 24.4)	234/11.3 (9.9 – 12.7)	643/31.1 (29.1 – 33.1)
	Not cancer related hosp. (n = 404)		6/1.5 (0.3 – 2.7)	9/2.2 (0.8 – 3.7)	14/3.5 (1.7 – 5.2)

Legend: *Not hospitalized in last month before death or patient dossier not reviewed. Hospital supplementary insurance status: no *HSI* = no hospital supplementary insurance, ECO = basic hospital supplementary insurance, *SP* + P = semi private and private hospital supplementary insurance (2 or single bed room).

### Descriptive results for the last 30 days before death

Among the 3,809 patients included there were slightly more male (52.7%) than female patients. Of the specified cancer diagnosis groups most patients were diagnosed with lung cancer (14.6%) followed by prostate cancer (10.4%, see Table 1). The mean age at death of all patients was 75.5 years.

A total of 2,608 (68.5%; 95% CI = 67.0-69.9) patients were hospitalized (see Table 2 for descriptive results). Of the hospitalized patients 80% (61%; 95% CI = 59.5-62.7 of all patients) died while in hospital. There was a difference in hospitalization frequency of almost 10% between male (72.9%, 95% CI = 71.0-74.9) and female patients (63.5%, 95% CI =61.3-65.7), and between patients with insurance type no HSI (63.0%, 95% CI = 60.3-65.7) or SP + P (73.6%, 95% CI = 71.0-76.3). Patients with lung cancer (76.1%, 95% CI = 72.6-79.7) had an almost 20% higher hospitalization frequency then patients with breast cancer (58.2%, 95% CI = 53.2-63.2). The canton with the highest hospitalization frequency was BS/BL (74.1%, 95% CI = 69.7-78.5) and the canton with the lowest frequency was VS (58.4%, 95% CI = 53.3-63.5).

In- and/or outpatient ACDT was given to 14.5% (95% CI = 13.4-15.6) of all patients. High proportions of ACDT use were seen in patients with lung cancer (20.3% (95% CI = 16.9-23.6), SP + P insured patients 20.5% (95% CI = 18.0-22.9), and in patients living in the canton TI (18.3% (95% CI = 15.8-20.7). In patients with a CRH ACDT use was 22.6% (95% CI = 20.8-24.4).

Overall 7.7% (95% CI = 6.7-8.4) of the patients received in- and/or outpatient RT. Of all cancer types lung cancer patients received the most RT (13.5%, 95% CI = 10.6-16.3). Of all cantons, TI was the one with the highest ACDT frequency, but had the lowest RT use (5.7%, 95% CI = 4.2-7.2).

The combined ACDT and/or RT use proportion was 20.3% (95% CI = 19.0-21.6). Far above this average figured lung cancer patients (31.1%, 95% CI = 27.2-34.9) and patients with insurance type SP + P (26.3%, 95% CI = 23.6-28.9). Much lower use was observed in patients living in the canton VS (13.2%, 95% CI = 9.7-16.7).

### Age effects

**Figure 2:**
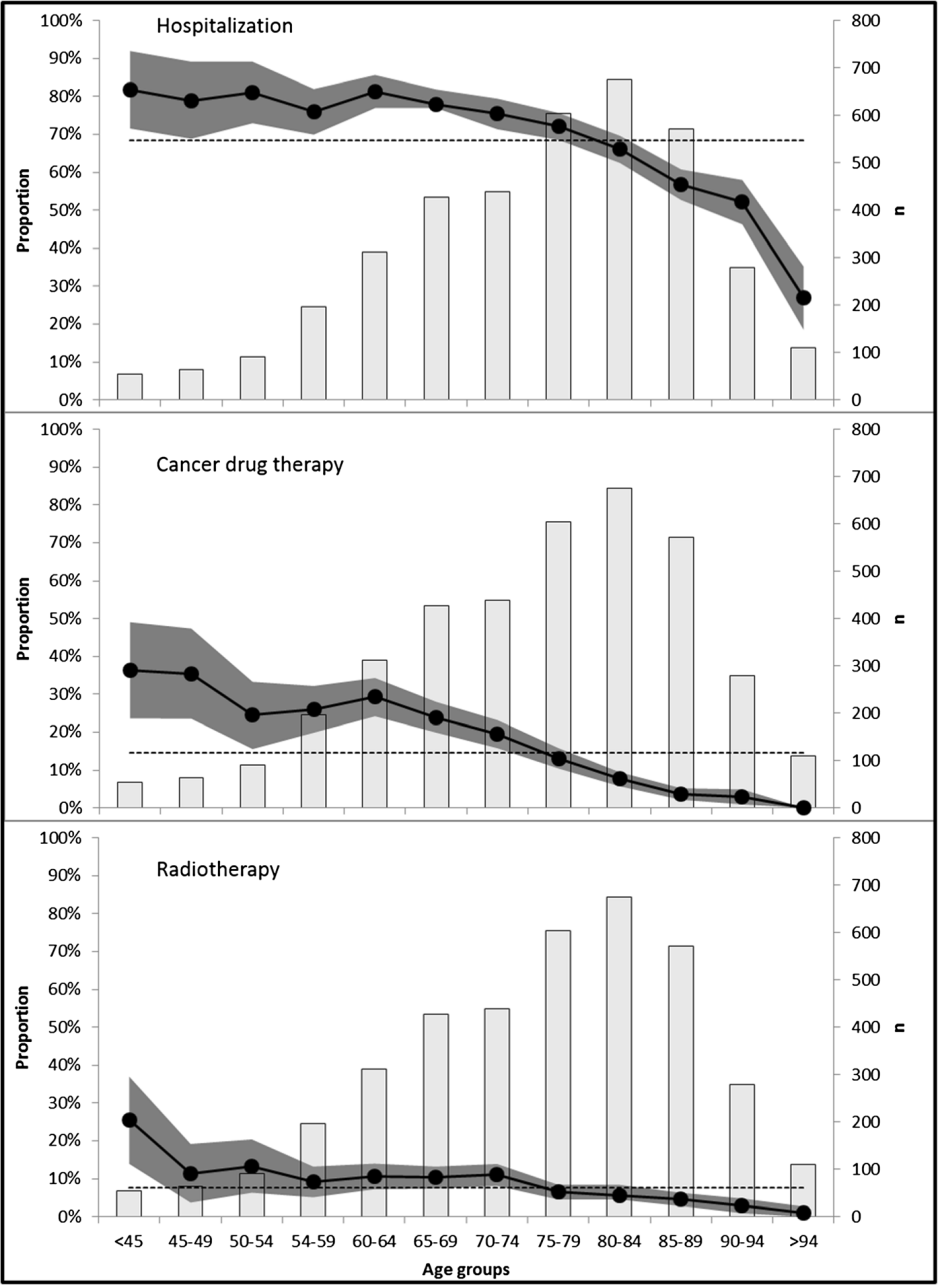
**Age effects on hospitalization and therapies.** Legend: •-• = proportion of patients with indication, dark grey area = 95% confidence interval on proportion, light grey bars = number of patients per age group, ---- = mean proportion across all age groups.

Hospitalization admission and ACDT use were strongly age-dependent; a linear decrease was observed after age 65 years. For RT a linear decrease was observed after the age of 75 (see Figure 2). Therefore, spline techniques were used in the multivariate logistic regression models to model the separate age effects for patients below or above 65 years old (models of hospitalization, ACDT use and ACDT and/or RT use) or for patients below or above 75 years old (model of RT use).

### Multivariable logistic regression

**Figure 3:**
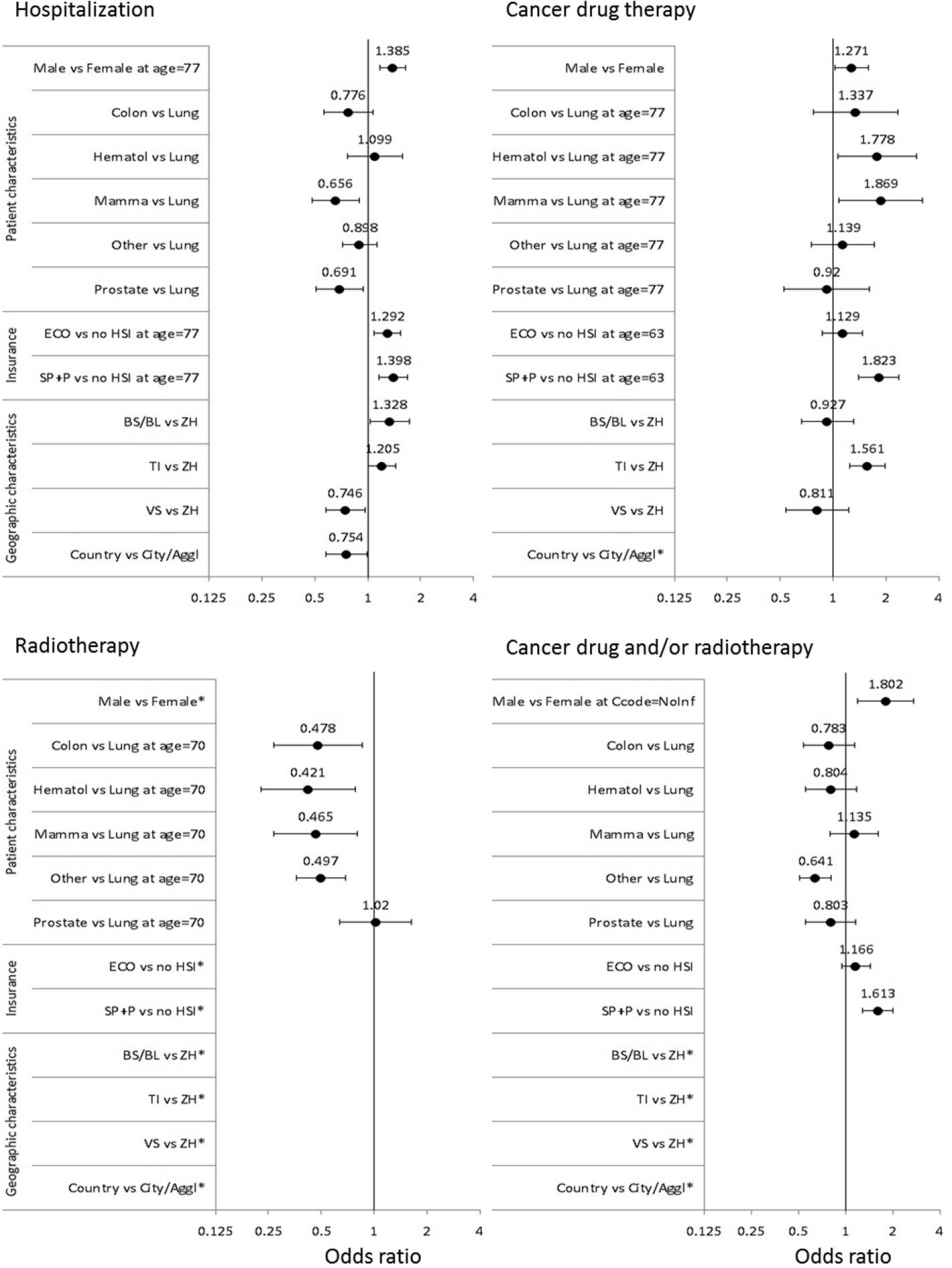
**Multivariate logistic regression.** Legend: * not significant > not included in the final multivariate model.

The multivariate logistic regression of hospitalization rates showed significant effects for the variables age, gender, cancer type, canton, borough type and insurance type (see Additional file 2: Table S2, and Figure 3). Males had a significantly higher odds of hospitalization than females (at age = 77, OR =1.38, 95% CI = 1.17-1.64). Compared to lung cancer patients, breast (OR = 0.66, 95% CI = 0.48-0.90) and prostate (OR = 0.69, 95% CI = 0.51-0.94) cancer patients were significantly less likely to be hospitalized. At the cantonal level BS/BL (OR = 1.38, 95% CI = 1.03-1.72) and TI (OR = 1.21, 95% CI = 1.01-1.44) showed significantly higher odds of hospitalization than ZH. In contrast VS (OR = 0.74, 95% CI = 0.58-0.97) and patients living in rural areas (OR = 0.75, 95% CI = 0.58-0.99) were less likely to be hospitalized. An OR of 1.40 (at age = 77, 95% CI = 1.16-1.69) was seen for patients with insurance type SP + P compared to patients without a HSI. There was significant interaction between age and gender, indicating that with increasing age the probability to be hospitalized decreased significantly more for female than male patients. In addition, significant interaction between age and insurance type implied that with increasing age the probability to be hospitalized decreased significantly more for ECO than for no HSI insured patients while the hospitalization probability for SP + P insured patients decreased (not significantly) less than for no HSI insured patients.

Receiving ACDT was significantly influenced by age, gender, cancer type, canton, and the control variable representing reason for hospitalization. The highest OR were seen for breast cancer (OR = 1.87, 95% CI = 1.08-3.22, at age = 77) compared to lung cancer patients, canton TI versus canton ZH (OR = 1.56, 95% CI = 1.24-2.00) and SP + P insured compared to no HSI (OR = 1.82, 95% CI = 1.40-2.38, at age = 63, see Figure 3). There was significant interaction between cancer type and age, indicating that for some cancer types (namely, hematologic and other cancers), with increasing age, the probability to receive ACDT decreased significantly less than for other cancer types. In addition, significant interaction between insurance type and age implied that in younger patients (<65 years) the probability to receive ACDT decreased significantly slower with increasing age for SP + P insured patients then no HSI insured patients, while this difference did no longer exist for older patients (>65 years).

Receiving RT was only significantly influenced by age and reason for hospitalization. Furthermore a significant interaction between cancer type and age was observed. At age = 70 breast (OR = 0.47, 95% CI = 0.27-0.81), colon (OR = 0.48, 95% CI = 0.27-0.85), hematological (OR = 0.42, 95% CI = 0.23-0.78) and other (OR = 0.50, 95% CI = 0.36-0.69) cancer patients received significantly less likely RT than lung cancer patients (see Figure 3). With increasing age the odds of receiving RT decreased less for lung cancer patients then all other patients.

For the endpoint of any cancer related therapy (ACDT and/or RT), cancer type and insurance type were significant predictors. Most prominent was the effect of insurance type with an OR of 1.61 (95% CI = 1.29-2.02) for SP + P insured compared to no HSI patients (see Figure 3). For this endpoint a significant interaction between gender and reason for hospitalization existed, implying that especially for CRH patients male had a higher probability to receive any type of cancer therapy.

## Discussion

Probabilities of hospitalization, ACDT use, RT use, and use of any cancer related therapy were generally higher in men than in women. Hospitalization frequency and therapy intensity decreased strongly with age. For hematologic (and other) cancers observed differences in proportions of ACDT and RT use depended more on age than for the remaining cancer types. Lung cancer patients received more ACDT and RT than all other patients while breast cancer patients at a higher age were the most likely to receive ACDT. In terms of cantonal differences, patients in canton TI were most likely to receive ACDT but least likely to receive RT. As a consequence, there was no significant difference between cantons in terms of administration of any cancer related therapy. For all endpoints, the canton VS generally showed the lowest use but only the probability of hospitalization was significantly lower than in ZH, the reference canton. Hospitalization frequencies were lower in rural areas. Insurance type had a strong effect on all endpoints; patients with insurance type SP + P were hospitalized significantly more often and were significantly more likely to receive cancer related therapies (ACDT, ACDT and/or RT).

The percentage of patients dying while hospitalized (61%) was much higher than percentages reported for Belgium and the Netherlands (29% and 19%, respectively, excluding patients suffering sudden death) [31] or the USA (38%) [32]. One reason for this finding may be a low availability of hospice care facilities in Switzerland. On the other hand, many acute-care hospitals have palliative care wards to which end-of-life patients may be transferred. We were not able to distinguish the types of wards where patients stayed and therefore the percentage of patients who died in a true acute-care setting may be substantially lower than 61%. Overall the observed percentage of patients receiving ACDT during the last month before death (14.5%) is in line with observations made by Earle et al. (USA, 14-18% in the last two weeks before death) [2]), Emanuel et al. (USA, 9% in the last month before death [6]) or Kao et al. (Australia, 18% within the last month before death [7]). The percentage patients receiving radiotherapy during the last month before death (7.7%) was also similar to the one observed by others [33,34].

Cantonal differences may be partially caused by diverse cultural attitudes of treating physicians as well as patients, which was also observed in other Swiss health services research studies [11-14]. The highest probabilities of hospitalization were seen for patients with a SP + P insurance. This result might hint at a financial incentive on the part of the care providers to hospitalize or to treat patients with a supplementary insurance status. On the other hand this increased utilization may be demand–driven by more demanding patients having paid for a more expensive insurance.

### Cancer type and age effects

Cancer type did influence several endpoints, with lung cancer patients being most likely to be hospitalized or to receive ACDT or RT. This finding is not surprising given the greater likelihood of lung cancer patients to experience dyspnea and other severe symptoms in their final disease stages [35]. Most of these differences were to be expected and are thought to be in accordance with the clinical practice for these cancers. The observed decrease in hospitalization probability and ACDT or RT use with increasing age was also to be expected and is in accordance with results from other studies [2,6].

Whether more or less frequent hospitalization or degree of ACDT/RT treatment are indicative of under- or over-treatment, and whether or not they are more strongly influenced by supply side or demand side factors, cannot be answered by this study. Unfortunately, we cannot differentiate between ACDT or RT given with a curative or a palliative intent. High use may indicate appropriate palliative care and should not primarily be interpreted as “aggressive” care, a term used by other authors [2,4,32,36]. Conversely, low use might indicate appropriate abstention from treatment but might as well hint at under-treatment. It is not possible to make the distinction with these data.

### Strengths and weakness

This study has some weaknesses. One limitation is that we have no reliable information on the cause of death for those patients not hospitalized during the last month before death. This may have led to an underestimation of ACDT and RT use due to the possibility of inclusion of some patients in the denominator who may not have had an active cancer disease at the time of death.

Another limitation is that the Helsana database may have had missing information on ACDT or RT use (for example missing ATC codes) which furthermore may lead to an underestimation of these outcomes. Also the information on HSI was only available for those patients who had this insurance with Helsana. For those patients with a supplementary insurance at another insurance company, this information was not available.

In addition, these findings are not generalizable to all of Switzerland for several reasons. The study is based on data from only one insurance company (albeit one of the largest in Switzerland with a market share of about 20%). Also Helsana on average serves an older population then the general Swiss population [11], and intensity of care decreases with age, so use of ACDT or RT in Switzerland may be higher than reported here. Furthermore data were available only from four out of 14 cantons with a cancer registry representing a small proportion of the national Swiss population. Especially the absence of data from purely French speaking cantons implies a potentially important knowledge gap, as these cantons represent a culturally distinct population with a different medical behavior and generally higher health care utilization [12].

The major strength of this study is that it provides an initial assessment of previously unavailable data and confirms a substantial degree of variation in end-of-life care for cancer patients in Switzerland. This implies a need for further research. The study also demonstrates the feasibility of Swiss research projects in the field of cancer-related Health Services Research that require linkage of data from different sources. It may serve as a model and may encourage further larger scale investigations in the field of cancer and other diseases, using similar approaches to combine several data sources. Next steps might be to implement data from more insurance companies as well as more cantons. In addition cost assessments may complement gained insight and help to better understand and guide the oncology community in providing cancer care at the end-of-life.

## Conclusion

This is the first larger scale Swiss study of patterns of care at the end-of-life of cancer patients. Data from four Swiss cantons show that the intensity of cancer specific care during the last month before death varies with age, cancer type, and place of residence as well as insurance type. The existence of such differences within a small country such as Switzerland may be partially driven by cultural differences on the side of physicians as well as patients and may be supported by the predominantly cantonal organization of the Swiss health care system. Conclusions regarding quality-of-care issues are not possible on the basis of this study, but would be of great importance in future research.

### Ethics

This study was approved by the ethics committees of the cantons Basel, Ticino, Valais and Zürich and the expert committee for data protection and professional secret in medical research of the federal office of health.

## Competing interests

The authors declared that they have no competing interest.

## Authors’ contributions

KMW designed the study, performed the data linkage, collected hospital data, performed the statistical analyses and drafted the manuscript. RA participated in the study design, helped with data collection in the hospitals and critically revised the manuscript. DK contributed to the statistical analyses and to critically revising the manuscript. MS, BP, TS participated in the study design, helped to coordinate data acquisition, made substantial contributions to interpretation of the data and critically revised the manuscript. RR, AB, SD, GJ, IK, KCG helped with the coordination of data acquisition and with the linkage. They critically revised the manuscript. All authors have read and approved the final manuscript.

## Pre-publication history

The pre-publication history for this paper can be accessed here:

http://www.biomedcentral.com/1471-2407/14/306/prepub

## Supplementary Material

Additional file 1: Table S1 Included anticancer drugs (ATC Codes). Click here for file

Additional file 2: Table S2 Multivariate logistic regression, estimates. Legend: * = P <0.05. CCode = Patient hospitalization/record information: CR = cancer related hospitalization, NCR = not cancer related hospitalization, NoInf = no information. Hospital supplementary insurance status: no HSI = no hospital supplementary insurance, ECO = basic hospital supplementary insurance, SP + P = semi private and private hospital supplementary insurance (2 or single bed room). NS/NI = not significant/not included in final model. Click here for file
