# Supplementary material for: Delivery of health care at the end of life in cancer patients of four swiss cantons: a retrospective database study (SAKK 89/09)
Source: BMC Cancer. 2014 May 1;14:306. doi: 10.1186/1471-2407-14-306 (PMC4101827; doi:10.1186/1471-2407-14-306)
Supplement: Additional file 1: Table S1 — Included anticancer drugs (ATC Codes). [file 1471-2407-14-306-S1.docx]

Supplementary Table I

Drug name ATC

| 6-Mercaptopurin | L01BB02 |
| --- | --- |
| ABX-EGF | L01XC08 |
| Adriblastin | L01DB01 |
| Afinitor | L01XE10 |
| AG013736 | None |
| Aldesleukin | L03AC01 |
| Alemtuzumab | L01XC04 |
| Alemtuzumabum | L01XC04 |
| Alimta | L01BA04 |
| Alkeran | L01AA03 |
| Amsacrin | L01XX01 |
| Amsacrinum | L01XX01 |
| Amsidyl | L01XX01 |
| Arsentrioxid | L01XX27 |
| Asparaginase | L01XX02 |
| Atriance | L01BB07 |
| Avastin | L01XC07 |
| Axitinib | None |
| Azacitidinum | L01BC07 |
| Bendamustin | L01AA09 |
| Bevacizumab | L01XC07 |
| Bevacizumabum | L01XC07 |
| Bleomycin | L01DC01 |
| Bleomycinum | L01DC01 |
| Bortezomib | L01XX32 |
| Caelyx | L01DB01 |
| Campto | L01XX19 |
| Capecitabin | L01BC06 |
| Capecitabinum | L01BC06 |
| Carboplatin | L01XA02 |
| Carboplatinum | L01XA02 |
| Carmubris | L01AD01 |
| Carmustin | L01AD01 |
| Ceenu | L01AD02 |
| Cerubidine | L01DB02 |
| Cetuximab | L01XC06 |
| Cetuximabum | L01XC06 |
| Chlorambucil | L01AA02 |
| Chlorambucilum | L01AA02 |
| Chlormethin | L01AA05 |
| Cisplatin | L01XA01 |
| Cisplatinum | L01XA01 |
| Cladribin | L01BB04 |
| Cladribinum | L01BB04 |
| Cosmegen | L01DA01 |
| Cyclophosphamid | L01AA01 |
| Cyclophosphamidum | L01AA01 |
| Cytarabin | L01BC01 |
| Cytarabinum | L01BC01 |
| Cytosar | L01BC01 |
| Dacarbazin | L01AX04 |
| Dacarbazinum | L01AX04 |
| Dacin | L01AX04 |
| Dacogen | L01BC08 |
| Dactinomycin | L01DA01 |
| Dasatinib | L01XE06 |
| Dasatinibum | L01XE06 |
| Daunomycin | L01DB02 |
| Daunorubicin | L01DB02 |
| Daunorubicinum | L01DB02 |
| Daunoxome | L01DB02 |
| Decitabine | L01BC08 |
| DepoCyte | L01BC01 |
| Detimedac | L01AX04 |
| Docetaxel | L01CD02 |
| Docetaxelum | L01CD02 |
| Doxorubicin | L01DB01 |
| Doxorubicini hydrochlorid | L01DB01 |
| DTIC Dome | L01AX04 |
| Efudix | L01BC02 |
| Eldisine | L01CA03 |
| Eloxatin | L01XA03 |
| Endoxan | L01AA01 |
| Epirubicin | L01DB03 |
| Epirubicini hydrochloridu | L01DB03 |
| Erbitux | L01XC06 |
| Erlotinib | L01XE03 |
| Erlotinibum | L01XE03 |
| Estracyt | L01XX11 |
| Estramustin | L01XX11 |
| Estramustini phosphas | L01XX11 |
| Etopophos | L01CB01 |
| Etoposid | L01CB01 |
| Etoposidum | L01CB01 |
| Everolimus | L01XE10 |
| Everolimus, RAD001 | L01XE10 |
| Everolimusum | L01XE10 |
| Farmorubicin | L01DB03 |
| Fludara | L01BB05 |
| Fludarabin | L01BB05 |
| Fludarabini phosphas | L01BB05 |
| FluoroUracil | L01BC02 |
| Fluorouracilum | L01BC02 |
| Gefitinib | L01XE02 |
| Gefitinibum | L01XE02 |
| Gemcitabin | L01BC05 |
| Gemtuzumab | L01XC05 |
| Gemzar | L01BC05 |
| Glivec | L01XE01 |
| Herceptin | L01XC03 |
| Holoxan | L01AA06 |
| Hycamtin | L01XX17 |
| Hydroxycarbamid | L01XX05 |
| Hydroxycarbamidum | L01XX05 |
| Ibritumomab tiuxetan | V10XX02 |
| Idarubicin | L01DB03 |
| Idarubicini hydrochloridu | L01DB06 |
| Ifosfamid | L01AA06 |
| Ifosfamidum | L01AA06 |
| Imatinib | L01XE01 |
| Imatinibum | L01XE01 |
| Interferon alfa-2a | L03AB04 |
| Interferon alfa-2b | L03AB05 |
| Interferonum alfa-2a ADNr | L03AB04 |
| Interferonum alfa-2b ADNr | L03AB05 |
| Intron A | L03AB05 |
| Iressa | L01XE02 |
| Irinotecan | L01XX19 |
| Irinotecani hydrochloridu | L01XX19 |
| Ixabepilon | L01DC04 |
| Ixempra | L01DC04 |
| Javlor | L01CA05 |
| Kidrolase | L01XX02 |
| Lanvis | L01BB03 |
| Lapatinib | L01XE07 |
| Lapatinibum | L01XE07 |
| Laromustin | L01AD02 |
| Lenalidomid | L04AX04 |
| Lenalidomidum | L04AX04 |
| Leukeran | L01AA02 |
| Leustatin | L01BB04 |
| Litak | L01BB04 |
| Litalir | L01XX05 |
| Lomustin | L01AD02 |
| Lomustinum | L01AD02 |
| Lysodren | L01XX13 |
| MabCampath | L01XC04 |
| Mabthera | L01XC02 |
| Melphalan | L01AA03 |
| Melphalanum | L01AA03 |
| Mercaptopurin | L01BB02 |
| Mercaptopurinum | L01BB02 |
| Methotrexat | L01BA01 |
| Methotrexate | L01BA01 |
| Methotrexatum | L01BA01 |
| Metoject | L01BA01 |
| Mitomycin | L01DC03 |
| Mitomycin C | L01DC03 |
| Mitomycinum | L01DC03 |
| Mitotane | L01XX13 |
| Mitoxantron | L01DB07 |
| Mitoxantronum | L01DB07 |
| Mustargen | L01AA05 |
| Mutamycin | L01DC03 |
| Myelotarg | L01XC05 |
| Natulan | L01XB01 |
| Navelbine | L01CA04 |
| Nelarabin | L01BB07 |
| Nelarabinum | L01BB07 |
| Nexavar | L01XE05 |
| Nilotinib | L01XE08 |
| Nilotinibum | L01XE08 |
| Nipent | L01XX08 |
| Novantron | L01DB07 |
| Oncaspar | L01XX02 |
| Oncovin | L01CA02 |
| Oxaliplatin | L01XA03 |
| Paclitaxel | L01CD01 |
| Paclitaxelum | L01CD01 |
| Panitumumab | L01XC08 |
| Panitumumabum | L01XC08 |
| Paraplatin | L01XA02 |
| Pemetrexed | L01BA04 |
| Pentostatin | L01XX08 |
| Pharmion | L04AX02 |
| Platiblastin S | L01XA01 |
| Platinol | L01XA01 |
| Prava | L01AD02 |
| Procarbazin | L01XB01 |
| Procarbazinum | L01XB01 |
| Proleukin | L03AC01 |
| Puri Nethol | L01BB02 |
| Raltitrexed, Paltitrexid | L01BA03 |
| Revlimid | L04AX04 |
| Ribomustin | L01AA09 |
| Rituximab | L01XC02 |
| Rituximabum | L01XC02 |
| Roferon A | L03AB04 |
| Sorafenib | L01XE05 |
| Sorafenibum | L01XE05 |
| Sprycel | L01XE06 |
| Streptozocin | L01AD04 |
| Sunitinib | L01XE04 |
| Sunitinibum | L01XE04 |
| Sutent | L01XE04 |
| Tarceva | L01XE03 |
| Tasigna | L01XE08 |
| Taxol | L01CD01 |
| Taxotere | L01CD02 |
| Temodal | L01AX03 |
| Temozolomid | L01AX03 |
| Temozolomidum | L01AX03 |
| Temsirolimus | L01XE09 |
| Temsirolimusum | L01XE09 |
| Teniposide | L01CB02 |
| Thalidomid | L04AX02 |
| Thiotepa | L01AC01 |
| Tioguanin | L01BB03 |
| Tioguaninum | L01BB03 |
| Tomudex | L01BA03 |
| Topotecan | L01XX17 |
| Topotecanum | L01XX17 |
| Torisel | L01XE09 |
| Trabectedin | L01CX01 |
| Trastuzumab | L01XC03 |
| Tretinoin | L01XX14 |
| Tretinoinum | L01XX14 |
| Trisenox | L01XX27 |
| Tyverb | L01XE07 |
| Vectibix | L01XC08 |
| Velbe | L01CA01 |
| Velcade | L01XX32 |
| Vepesid | L01CB01 |
| Vesanoid | L01XX14 |
| Vidaza | L01BC07 |
| Vinblastin | L01CA01 |
| Vincristin | L01CA02 |
| Vincristini sulfas | L01CA02 |
| Vindesin | L01CA03 |
| Vinflunin | L01CA05 |
| Vinorelbin | L01CA04 |
| Vinorelbinum | L01CA04 |
| Vumon | L01CB02 |
| Xeloda | L01BC06 |
| Yondelis | L01CX01 |
| Zanosar | L01AD04 |
| Zavedos | L01DB06 |
| Zevalin | V10XX02 |
