# Supplementary material for: Delivery of health care at the end of life in cancer patients of four swiss cantons: a retrospective database study (SAKK 89/09)
Source: BMC Cancer. 2014 May 1;14:306. doi: 10.1186/1471-2407-14-306 (PMC4101827; doi:10.1186/1471-2407-14-306)
Supplement: Additional file 2: Table S2 — Multivariate logistic regression, estimates. Legend: * = P <0.05. CCode = Patient hospitalization/record information: CR = cancer related hospitalization, NCR = not cancer related hospitalization, NoInf = no information. Hospital supplementary insurance status: no HSI = no hospital supplementary insurance, ECO = basic hospital supplementary insurance, SP + P = semi private and private hospital supplementary insurance (2 or single bed room). NS/NI = not significant/not included in final model. [file 1471-2407-14-306-S2.docx]

Supplementary Table II: Multivariable logistic regression results

|  | **Hospitalization** | | **Cancer drug therapy** | | **Radiotherapy** | | **Cancer drug- and or radiotherapy** | |
| --- | --- | --- | --- | --- | --- | --- | --- | --- |
|  | estimate (95%CI) | *P*-Value | estimate (95%CI) | *P* -Value | estimate (95%CI) | *P* -Value | estimate (95%CI) | *P* -Value |
| **Intercept** | 1.14  (-0.08 ; 2.35) |  | 0.69  (-1.05 ; 2.42) |  | -1.99  (-4.07 ; 0.09) |  | 0.73  (-0.34 ; 1.81) |  |
| **Patient hospitalization/chart information:**  reference category: cancer-related hospitalisation |  | |  | <0.0001 |  | <.0001 |  | <.0001 |
| No cancer-related hospitalisation |  |  | -2.46  (-3.28 ; -1.64)* | <.0001 | -1.45  (-2.13 ; -0.76)* | <.0001 | -1.45  (-2.19 ; 0.71)* | 0.0001 |
| No information |  |  | -1.21  (-1.47 ; -0.95)* | <.0001 | -1.12  ( -1.46 ; -0.78)* | <.0001 | -1.52  (-1.87 ; -1.18)* | <.0001 |
| **Age – spline 1, first part:**  if age ≤65, multiply with true age,  if age>65, multiply with 65 | 0.002  (-0.02 ; 0.02) | 0.8174 | -0.02  (-0.05 ; 0.004) | 0.0965 |  | | -0.02  (-0.03 ; 0.00) | 0.0550 |
| **Age - spline 1, second part:**  if age ≤65, multiply with 0  if age >65, multiply with true age - 65 | -0.06  (-0.07 ; -0.04)* | <0.0001 | -0.12  (-0.16 ; -0.08)* | <0.0001 |  |  | -0.06  ( -0.07 ; -0.05)* | <.0001 |
| **Age – spline 2, first part:**  if age ≤75, multiply with true age,  if age>75, multiply with 75 |  | |  | | 0.01  (-0.02 ; 0.04) | 0.6525 |  | |
| **Age – spline 2, second part:**  if age ≤75, multiply with 0  if age >75, multiply with true age - 75 |  |  |  |  | -0.03  (-0.06 ; 0.004)* | 0.0265 |  |  |
| **Gender:**  male versus female | 0.04  (-0.23 ; 0.31) | 0.7692 | 0.24  (0.02 ; 0.46)* | 0.0340 | NS/NI | | 0.19  (-0.03 ; 0.40) | 0.0861 |
| **Cancer diagnosis:**  reference category: lung cancer |  | 0.007 |  | 0.0008 |  | 0.0696 |  | 0.0004 |
| Colon | -0.25  (-0.57 ; 0.07) | 0.1216 | -0.06  (-0.63 ; 0.50) | 0.8245 | 3.37  (-0.16 ; 6.90) | 0.0611 | -0.24  (-0.62 ; 0.13) | 0.1960 |
| Hematologic | 0.09  (-0.26 ; 0.45) | 0.6029 | -0.33  (-0.91 ; 0.25) | 0.2630 | 2.19  (-1.01 ; -5.39) | 0.1802 | -0.22  (-0.60 ; 0.16) | 0.2584 |
| Breast | -0.43  (-0.74 - -0.11)* | 0.0079 | 0.35  (-0.14 ; 0.84) | 0.1579 | 3.20  (-0.18 ; 6.59) | 0.0634 | 0.13  (-0.23 ; 0.48) | 0.4838 |
| Other | -0.11  (-0.33 ; 0.12) | 0.3517 | -0.53  (-0.53 ; -0.19)* | 0.0024 | 1.06  (-1.43 ; 3.54) | 0.4060 | -0.44  (-0.67 ; -0.21)* | 0.0002 |
| Prostate | -0.37  (-0.68 ; -0.06)* | 0.0182 | -0.03  (-0.74 ; 0.69) | 0.9451 | 7.03  (1.46 ; 12.59)* | 0.0133 | -0.22  (-0.59 ; 0.15) | 0.2387 |
| **Insurance type:**  reference category: no supplementary health insurance |  | 0.003 |  | 0.0979 | NS/NI | |  | <.0001 |
| Basic hospital supplementary insurance (ECO) | 0.54  (0.23 ; 0.85)* | 0.0006 | 0.12  (-2.15 ; 2.39) | 0.9177 |  |  | 0.15  (-0.06 ; 0.37) | 0.1625 |
| Semi private and private hospital supplementary insurance (SP + P) | 0.28  (-0.04 ; 0.60) | 0.0882 | -2.96  (-5.97 ; 0.05) | 0.0539 |  |  | 0.48  (0.25 ; 0.70)* | <.0001 |
| **Borough type**  rural versus city / agglomeration | -0.28  (-0.55 ; -0.01)* | 0.0410 | NS/NI | | NS/NI | | NS/NI | |
| **Canton of residence:**  reference category: Zürich (ZH) |  | 0.0009 |  | 0.0003 | NS/NI | | NS/NI | |
| Basel (BS/BL) | 0.28  (0.03 ; 0.54)* | 0.0307 | -0.08  (-0.42 ; 0.27) | 0.6672 |  |  |  |  |
| Ticino (TI) | 0.19  (0.01 ; 0.36)* | 0.0410 | 0.45  (0.22 ; 0.67)* | 0.0001 |  |  |  |  |
| Valais (VS) | -0.30  (-0.55 ; -0.04)* | 0.0257 | -0.21  (-0.63 ; 0.21) | 0.3235 |  |  |  |  |
| **Interactions** | | | | | | | | |
| **Age - spline 1, second part * gender:**  If age ≤65, multiply with 0,  if age >65, multiply with true age-65 |  | 0.0037 |  | |  | |  | |
| iIf male | 0.02  (0.01 ; 0.04)* | 0.0037 |  |  |  |  |  |  |
| **Age - spline 1, second part * insurance type:**  If age ≤65, multiply with 0,  if age >65, multiply with true age-65 |  | 0.0097 |  |  |  |  |  |  |
| If ECO | -0.02  (-0.04 ; -0.01)* | 0.0108 |  |  |  |  |  |  |
| If SP + P | 0.005  (-0.02 ; 0.02) | 0.6548 |  |  |  |  |  |  |
| **Age - spline 1, first part * insurance type:**  if age ≤65, multiply with true age,  if age>65, multiply with 65 |  | |  | 0.0419 |  |  |  |  |
| If ECO |  |  | 0.00  (-0.04 ; 0.04) | 0.9990 |  |  |  |  |
| If SP + P |  |  | 0.06  (0.01 ; 0.10)* | 0.0220 |  |  |  |  |
| **Age - spline 1, second part *cancer type:**  if age ≤65, multiply with 0,  if age >65, multiply with true age-65 |  | |  | 0.0216 |  | |  | |
| If colon |  |  | 0.029  (-0.03 ; 0.09) | 0.3321 |  |  |  |  |
| If hematologic |  |  | 0.08  (0.02 ; 0.13)* | 0.0085 |  |  |  |  |
| If breast |  |  | 0.02  (-0.03 ; 0.08) | 0.4162 |  |  |  |  |
| If other |  |  | 0.05  (0.01 ; 0.10)* | 0.0131 |  |  |  |  |
| If prostate |  |  | -0.005  (-0.07 ; 0.06) | 0.8866 |  |  |  |  |
| **Age – spline 2, second part * cancer type:**  if age ≤75, multiply with 0,  if age >75, multiply with true age-75 |  |  |  | |  | 0.0437 |  |  |
| If colon |  |  |  |  | -0.58  (-0.11 ; -0.006)* | 0.0288 |  |  |
| If hematologic |  |  |  |  | -0.04  (-0.09 ; 0.004) | 0.0749 |  |  |
| If breast |  |  |  |  | -0.06  (-0.11 ; -0.006)* | 0.0297 |  |  |
| If other |  |  |  |  | -0.03  (-0.06 ; 0.01) | 0.1792 |  |  |
| If prostate |  |  |  |  | -0.10  (-0.18 ; -0.02)* | 0.0122 |  |  |
| **Gender * patient hospitalization/chart information** |  |  |  |  |  | |  | 0.0186 |
| If male and no cancer-related hospitalisation |  |  |  |  |  |  | -1.15  (-2.26 ; -0.04)* | 0.0416 |
| If male and no information |  |  |  |  |  |  | 0.40  (-0.04 ; 0.85) | 0.0755 |

Legend: *= *P* <0.05, ECO = basic hospital supplementary insurance, SP + P= semi private and private hospital supplementary insurance (double or single bed room), NS/NI = not significant / not included in final model.
